# Morphological Diversity and Preliminary DNA Barcoding of *Xylaria* (Xylariales) from Estación Científica San Francisco, Including *Xylaria aenea* as a New Record for Ecuador

**DOI:** 10.3390/jof12030211

**Published:** 2026-03-15

**Authors:** Darío Cruz, Juan Pablo Suárez, Andres Chamba, Paola Duque-Sarango, Luisa Espinosa, Roo Vandregrift

**Affiliations:** 1 Microbial Systems Ecology and Evolution Research Group, Department of Biological Sciences, Biology School, Universidad Técnica Particular de Loja, San Cayetano Alto s/n, Loja 110107, Ecuador; jpsuarez@utpl.edu.ec (J.P.S.); crystand_01@hotmail.es (A.C.); ldespinosa2@utpl.edu.ec (L.E.); 2 Instituto Nacional de Biodiversidad INABIO, Quito 170135, Ecuador; werdnus@gmail.com; 3 Research Group Recursos Hídricos (GIRH-UPS), Universidad Politécnica Salesiana, El Vecino Campus, Calle Vieja 12-30 y Elia Liut, Cuenca 010105, Ecuador; pduque@ups.edu.ec; 4 Institute of Ecology and Evolution, University of Oregon, 272 Onyx Bridge, 5289 University of Oregon, Eugene, OR 97403-5289, USA

**Keywords:** Ascomycota, fungal diversity, ITS-5.8S, LSU, Sordariomycetes, tropical rain forest

## Abstract

The genus *Xylaria* comprises numerous species, particularly prevalent in tropical ecosystems such as those of Ecuador. Despite its ecological importance, the taxonomy of the genus remains challenging, and much of its diversity in the Neotropics remains under-documented. This study provides a preliminary characterization of the *Xylaria* diversity at the Estación Científica San Francisco, an Andean biodiversity hotspot in Southern Ecuador. Through an integrated approach including detailed macro- and micro-morphological descriptions and nuclear ribosomal DNA (nrDNA ITS and LSU) phylogenetic analyses, 20 *Xylaria* specimens were examined. As a result, ten species were recognized: *Xylaria adscendens*, *X.* cf. *anisopleura*, *X. apiculata*, *X. curta*, *X. enterogena*, *X. fissilis*, *X. globosa*, *X. aff. telfairii*, *X. tuberoides*, and *X. aenea*, the latter representing a new record for Ecuador. The phylogenetic analysis presented here serves as a preliminary systematic positioning of these specimens within the genus rather than a comprehensive global reconstruction. While these ribosomal markers provided preliminary insights into species relationships, partial incongruence with morphospecies highlights the evolutionary complexity of certain lineages and underscores the need for future multilocus studies. Furthermore, four additional phylotypes found in their anamorphic state are documented, suggesting that local diversity exceeds current records. By providing detailed morphological documentation supported by preliminary barcode data from a poorly sampled region, this study contributes vital information to the global understanding of *Xylaria* and underscores the importance of Southern Ecuador as a reservoir of fungal diversity.

## 1. Introduction

*Xylaria* Hill ex Schrank is the largest genus within the family Xylariaceae Tul. & C. Tul., with 901 epithets deposited in Mycobank (accessed: 8 July 2024). Ecologically, species of *Xylaria* are recognized as decomposers of organic matter [1,2], particularly lignin- and cellulose-rich wood [3,4,5]. Morphologically, the genus is characterized by having perithecia embedded in sometimes branched stromata, which are commonly carbonaceous, cracked, and rough. In their immature stage, the stromata typically produce white conidia along their upper portions. In some species, such as those within the *Xylaria polymorpha* complex [6], including *X. anisopleura*, *X. bulbosa*, *X. feejeensis*, *X. longipes*, *X. obovate*, *X. polymorpha*, *X. schweinitzii*, and *X. tuberiformis*, these conidial structures may later be replaced by perithecia during stromatal maturation [6,7,8]. Microscopically, *Xylaria* have unitunicate and inoperculate cylindrical asci with an amyloid apical apparatus, generally with eight ascospores in each. The ascospores are typically pigmented, ranging from pale brown to black, ellipsoid-inequilateral in shape, and typically having a hyaline germ slit [7,8].

The genus exhibits a high degree of intraspecific variability and interspecific similarity, which makes it difficult to identify taxonomically informative characters [9]. This has contributed to significant confusion in historical taxonomy [10,11]. Due to this morphological complexity, species identification is most effectively achieved using integrated morphological and molecular datasets. In this approach, morphological data are validated through phylogenetic analysis using a combination of genetic markers. Essential markers include the barcode marker nrDNA ITS-5.8S and LSU [12,13,14,15,16,17,18]. Other useful markers include α-actin, β-tubulin, and RNA polymerase subunit II [19,20].

Species of *Xylaria* have a global distribution, often reported as saprotrophs in tropical and subtropical regions with varying levels of endemism [21,22,23]. This significant group of fungi remains understudied, particularly in South America. Notable studies are limited to a few countries, including Argentina [24,25,26], Colombia [27], Brazil [28,29,30], and Venezuela [31]. Although Ecuador is a mega-diverse country, there are few relevant studies on fungi across its different ecosystems [32,33,34].

To address this knowledge gap, we conducted a mycological survey of *Xylaria* species at the Estación Científica San Francisco, a site located within the Andean biodiversity hotspot [35]. Currently, mycorrhizal fungi are the only group of fungi from this site that have been studied in depth, using both molecular [36,37,38] and morphological methods [39]. No collection-based studies on macrofungi have been conducted at the Estación Científica San Francisco to date. Therefore, this study aims to provide an important contribution to the knowledge of fungal diversity in Ecuador by performing a morphological and molecular characterization of *Xylaria* specimens collected from this forest reserve in southern Ecuador.

## 2. Materials and Methods

### 2.1. Collecting and Site Description

Sampling of *Xylaria* specimens was carried out at Estación Científica San Francisco, a tropical mountain forest reserve bordering Podocarpus National Park (PNP) in Zamora Chinchipe province. Detailed information about the forest is given in [40]. The ascomata were collected between April 2014 and October 2015 from the paths named AT and T2, at elevations of approximately 1900 and 2500 m a.s.l. respectively. Vouchered specimens were deposited in the fungarium of Universidad Técnica Particular de Loja’s Herbarium [HUTPL(F)] with accession codes provided in Table 1.

### 2.2. Morphological Diagnosis

We recorded the observable characteristics of the ascomata, including color, texture, and measurements of stipe and stroma length and width. To examine internal structures, we made freehand horizontal and transversal sections of each stroma to observe the color of the perithecia and internal tissues. For microscopic analysis, fresh and dried samples of perithecial contents were stained with Melzer’s reagent to test for an amyloid reaction. Other reagents, such as 1% Phloxine, 1% Methyl blue, and 3% Potassium hydroxide (KOH), were also used as needed. All sections were examined at 1000x magnification using an Olympus BX51 light microscope (Olympus Corporation, Tokyo, Japan).

The length and width of various microscopic structures were measured using iWork EX image analysis software (version 2.0), which was integrated with a Lanoptik-MDX503 digital microscopy camera (Lanoptik Ltd., Fuzhou, China). We took at least 25 measurements for perithecia and asci, and at least 30 measurements for ascospores. Ascospore measurements are presented as a range (min–) with the mean (–max) and the Q-value (average spore length/width ratio) [41]. We classified ascospore shapes primarily according to [42].

Species were identified using scientific literature and standard taxonomic keys, which are cited in the corresponding species descriptions. The term “cf.” (confer) indicates that specimens are morphologically similar to a specific species but have an uncertain phylogenetic relationship at the species level. The term “aff.” (affinis) indicates that a described species resembles its holotype but is considered distinct and potentially undescribed.

### 2.3. PCR Amplification and Sequencing of DNA

PCR was performed using the Phire^®^ Plant Direct PCR Kit (Thermo Scientific, Waltham, MA, USA) following the manufacturer’s instructions. A small piece of stroma, approximately 1 mm^3^ in size and containing perithecia when possible, was placed into 20 μL of Dilution Buffer. Of this solution, 2 μL were used as the PCR template. The total PCR reaction volume was 20 μL, including 0.4 μL (25 pmol) of each primer and 0.4 μL of bovine serum albumin (BSA 10%) as an additive. For amplification, we used a nested PCR approach. The first reaction utilized the universal primers ITS1-F [43] and LR5 [44]. A subsequent, nested PCR was then performed using the primers ITS1 [45] and NL4 [46]. The PCR conditions for both reactions were as follows: an initial denaturation at 98 °C for 5 s, followed by 40 cycles. Each cycle consisted of denaturation at 98 °C for 10 s, annealing at 55 °C for 20 s, and extension at 72 °C for 30 s. A final extension at 72 °C for 10 min was performed to complete the reaction. PCR products were visualized on a 1% (*w*/*v*) agarose gel stained with 1X GelRed™ Safe Nucleic Acid Gel Stain (Biotium, Hayward, CA, USA).

All positive PCR products (15–18 μL) were purified by adding an equal volume (1:1 ratio) of PEG (20% Polyethylene glycol 8000/2.5 M NaCl), followed by incubation at 37 °C for 15 min. The mixture was then centrifuged at 16,500 RCF for 15 min. The resulting pellets were rinsed with 80% cold ethanol and dried for 45 min at room temperature under a sterile flow hood. Finally, the dried pellets were re-suspended in 60 μL of deionized water. The purified PCR products, at a final concentration of 20–40 ng/μL, were sent to Macrogen (Seoul, Republic of Korea) for sequencing.

### 2.4. Phylogenetic Analyses

Sequence chromatograms were verified using Codon Code Aligner 5.1.4 (CodonCode Corporation, Centerville, MA, USA). The resulting sequences were aligned with reference sequences from the NCBI GenBank (https://www.ncbi.nlm.nih.gov) and UNITE (https://unite.ut.ee) databases (accessed: 10 January 2026). Sequences showing at least 95% similarity, as well as those containing taxonomic information such as type material or species names, were incorporated into the analysis (Table 2).

All nrITS-5.8S and LSU D1/D2 sequences were aligned using the G-INS-i strategy in MAFFT v.7 [47]. The ITS-5.8S region of the sequences was defined and extracted using ITSx v.1.0.11 [48], while the LSU D1/D2 region was empirically defined by its length within the alignment against sequences with known gene notation (e.g., AY373291, AJ313443, AY373294).

Phylogenetic trees were constructed separately for each region (ITS-5.8S and LSU D1/D2). Maximum Likelihood (ML) phylogenetic trees were built using MEGA 11 [49] with 1000 bootstrap replicates [50] and the GTR+G+I DNA substitution model [51]. Bayesian Posterior Probability (BPP) analysis was performed using the GTR+I+G substitution model [52]. Two independent runs of four Markov chains each were run for 4 million generations with random starting trees. Trees were sampled every 100 generations, and a 50% majority-rule consensus tree was generated from the final 24,000 trees. Convergence and effective sample sizes (ESS) were assessed with Tracer v1.5 [53].

The trees presented represent the best-scoring topologies inferred from the ML analysis. Node support values are indicated as ML bootstrap percentages (BS) and Bayesian posterior probabilities (BPP) from the MrBayes analysis. The sequences generated in this study are available in GenBank under accession numbers OR086091–OR086094 and OR088119–OR088134.

**Table 2:** *Xylaria* sequences included in the phylogenetic analysis, with accession numbers and associated published sources.

Fungarium or Strain Code	Determination	ITS-5.8S (Accession Numbers)	Source	References
Strain 570 (HAST, JF)	*Xylaria adscedens*	GU300101	From fruitbody	[20]
Strain 1114	*Xylaria adscedens*	KP133298	From fruitbody	[33]
Strain 865 (JDR)	*Xylaria adscedens*	GU322432	From fruitbody	[20]
Strain PR7	*Xylaria globosa*	AY909007	From fruitbody	[13]
Strain INBio:261A	*Xylaria adscedens*	KR534704	Endophyte in *Smilax panamensis*	[54]
Strain E63	*Xylaria multiplex*	JQ814313	Endophyte in *Hevea brasiliensis*	[55]
Strain MP748	*Xylaria brevipes*	KJ154952	From fruitbody	Vietnam (Unpublished)
Voucher 15223	*Xylaria curta*	JF908804	Fruitbody	[56]
Strain 938	*Xylaria anisopleura*	KP133317	From fruitbody	[33]
Strain 1074	*Xylaria anisopleura*	KP133318	From fruitbody	[33]
Strain 1089	*Xylaria apiculata*	KP133325	From fruitbody	[33]
Strain 1062	*Xylaria apiculata*	KP133335	From fruitbody	[33]
Strain 1090	*Xylaria apiculata*	KP133337	From fruitbody	[33]
Strain 94080508 (HAST)	*Xylaria venosula*	EF026149	From “twigs”	[57]
Strain SCAU-F-161	*Xylaria apiculata*	KF881786	Endophyte in *Melia toosendan*	[58]
Strin 89041211 (HAST)	*Xylaria arbuscula*	GU300090	From “bark”	[20]
Strain 205 (WSP:205)	*Xylaria bambusicola*	NR_153200	From **Holotype**	[20]
Strain 494 (HAST, JF)	*Xylaria curta*	GU322444	From fruitbody	[20]
Strain 92092022 (HAST)	*Xylaria curta*	GU322443	From fruitbody	[20]
QCAM4545	*Xylaria* sp.	MG768837	Fruitbody	[59]
Strain 897	*Xylaria curta*	KP133352	From fruitbody	[33]
Strain R1	*Xylaria curta*	KP133356	From fruitbody	[33]
Strain Vega421	Xylariaceae sp.	EU009999	Endophyte in *Coffea arabica*	Colombia (Unpublished)
Strain C227	*Xylaria curta*	MK304420	Endophyte in *Ageratina adenophora*	[60]
BCC 1067	*Xylaria* sp.	DQ139271	Fruitbody	[61]
Strain 1027	*Xylaria cuneata*	KP133351	From fruitbody	[33]
Strain 917	*Xylaria enterogena*	KP133371	From fruitbody	[33]
Strain 1049	*Xylaria enterogena*	KP133372	From fruitbody	[33]
Strain 785 (HAST, JF)	*Xylaria enterogena*	GU324736	From fruitbody	[20]
Strain 987	*Xylaria enterogena*	KP133368	From fruitbody	[33]
Strain 1053	*Xylaria* aff. *comosa*	KP133304	From fruitbody	[33]
Robert L. Gilbertson Mycological Herbarium 1371	*Xylaria* sp.	KT289542	Endolichenic in *Peltigera neopolydactyla*	[62]
Robert L. Gilbertson Mycological Herbarium 1364	*Xylaria* sp.	KT289541	Endolichenic in *Peltigera neopolydactyla*	[62]
E13415E	*Xylaria* sp.	KF466899	Endophyte in *Costus laevis*	Ecuador (Unpublished)
Strain 367 (HAST, JF)	*Xylaria fissilis*	GU300073	From fruitbody	[20]
Strain 775 (HAST, JF)	*Xylaria globosa*	GU324735	From fruitbody	[20]
Strain 933	*Xylaria globosa*	KP133428	From fruitbody	[33]
Strain 979	*Xylaria globosa*	KP133429	From fruitbody	[33]
Strain 995	*Xylaria globosa*	KP133345	From fruitbody	[33]
Strain 976	*Xylaria globosa*	KP133426	From fruitbody	[33]
INBio:2303C	*Xylaria schweinitzii*	KR534679	Endophyte in plants	[54]
UOC MINNP MK33b	*Xylaria schweinitzii*	KT037020	From fruitbody	Sri Lankan (Unpublished)
Strain 421 (HAST, JF)	*Xylaria telfairii*	GU324737	From fruitbody	[20]
Strain 90081901 (HAST)	*Xylaria telfairii*	GU324738	From fruitbody	[20]
TSJ845	*Xylaria telfairii*	KF937370	Fruitbody	[63]
Strain 987	*Xylaria telfairii*	KP133368	From fruitbody	[33]
GAB193	*Xylaria longipes*	KY250409	Fruitbody	Gabon (Unpublished)
Strain CBS 14873	*Xylaria longipes*	AY909013	From fruitbody	[13]
Strain R22	*Xylaria tuberorides*	KP133545	From fruitbody	[33]
Strain 475 (HAST, JF)	*Xylaria tuberorides*	GU300074	From fruitbody	[20]
Hao & Guo & Han 060203	*Xylaria xanthinovelutina*	MH425284	From fruitbody	China (Unpublished)
Strain 553 (HAST, JF)	*Xylaria ianthinovelutina*	GU322441	From fruitbody	[20]
Strain 189 (JDR)	*Xylaria culleniae*	GU322442	From fruitbody	[20]
Strain C24	*Xylaria ianthinovelutina*	JQ936302	Endophyte in soybean	[64]
Strain 945	*Xylaria* aff. *comosa*	KP133303	From fruitbody	[33]
Strain R0	*Xylaria* aff. *comosa*	KP133308	From fruitbody	[33]
Strain 901	*Xylaria* aff. *comosa*	KP133307	From fruitbody	[33]
Strain 860 (JDR)	*Xylaria cubensis*	GU991523	From fruitbody	[20]
NR-2006-D65	*Xylaria* sp.	DQ480358	Endophyte in *Garcinia* plants	[65]
STRI:ICBG-Panama:TK985	Fungal endophyte	KF436104	Endophyte in *Saccharum* sp.	[66]
Strain F1124	Xylariaceae sp.	KU747729	Endophyte in *Campyloneurum serpentinum*	[67]
Strain ATCC 58729	*Parahypoxylon papillatum*	NR_155153	from **epitype** of *Hypoxylon papillatum*	[68]
Strain CHTAE15	*Xylaria curta*	JF773598	Endophyte in *Taxus globosa*	[69]
STRI:ICBG-Panama:TK985	Fungal endophyte	KF436104	Endophyte in *Saccharum* sp.	[66]
Strain CBS 385.35	*Xylaria mali*	MH867225	From fruitbody	[70]
Strain CBS 162 22	*Xylaria polymorpha*	MH866242	From fruitbody	[70]
Strain BCC 22966	*Xylaria papulis*	AB376811	Endophyte on wood	[71]
Strain DSM 110363	*Xylaria multiplex*	MN833802	Deciduous deadwood	[72]
Strain C4015B2SNA2CC460	*Xylaria hypoxylon*	KP143687	From marine sponge	[73]
Strain FL0490	*Xylaria venustula*	JQ760209	Endolichenic in *Cladonia didyma*	[74]
Strain BCC 1085	*Xylaria coccophora*	AB376688	Endophyte/saprobe on wood	[71]
Strain FL0512	*Xylaria* sp.	JQ760231	Endophyte in *Usnea mutabilis*	[74]
Strain 931	*Xylaria* sp.	KP133516	From fruitbody	[33]
Strain BCC 17352	*Xylaria anisopleura*	AB376732	Endophyte/saprobe on wood	[71]
Strain 1m_VC1	*Xylaria* cf. *microceras*	KT250979	From fruitbody	[62]
MFLUCC 11-0606	*Xylaria bambusicola*	KU863148	Fruitbody	[67]
Strain BCC 1086	*Xylaria juruensis*	AB376689	Endophyte/saprobe on wood	[71]
Strain FL1283	*Xylaria arbuscula*	JQ760898	Endolichenic in *Cladonia leporina*	[74]
Strain CBS 126416	*Xylaria arbuscula*	MH875561	From fruitbody	[70]
Strain BCC 1083	*Xylaria juruensis*	AB376687	Endophyte/saprobe on wood	[71]
Strain B1A0816P30CC497	Xylariaceae sp.	KP306978	From marine sponge	[73]
STRI:ICBG-Panama:TK36	Fungal endophyte	KF435419	Endophyte in *Cordia lasiocalyx*	[66]
Strain BCC 1151	*Xylaria curta*	AB376702	Endophyte/saprobe on wood	[71]
Strain BCC 1115	*Xylaria feejeensis*	AB376696	Endophyte/saprobe on wood	[71]
Strain CBS 128357	*Xylaria enteroleuca*	MH876349	From fruitbody	[70]
Strain CHTAR111	*Xylaria cubensis*	GU048579	Endophyte in *Taxus globosa*	[69]
Strain NC1163	*Xylaria* cf. *heliscus*	JQ761807	Endophyte in *Tsuga canadensis*	[74]
Strain FL1767 (ARIZ)	*Xylaria* cf. *heliscus*	KU683899	Endophyte in *Quercus inopina*	[67]
Strain FL1161	*Xylaria* cf. *heliscus*	JQ760778	Endophyte in *Parmotrema rampoddense*	[74]
Strain B1a0283EM2CC388	*Xylaria* sp.	KP306964	From marine sponge	[73]
TSJ845	*Xylaria telfairii*	KJ130993	Fruitbody	Unpublished
Strain FL1777 (ARIZ)	*Xylaria* sp.	KU683903	Endophyte in *Quercus inopina*	[67]
Strain F1825	*Xylaria* sp.	KU747819	Endophyte in fern frond	[67]
Strain BCC 18361	*Xylaria tuberorides*	AB376736	Endophyte/saprobe on wood	[71]
Strain FL0632 (ARIZ)	*Xylaria cubensis*	JQ760340	Endolichenic in *Parmotrema tinctorum*	[74]
Strain NC1232	*Xylaria cubensis*	JQ761869	Endophyte in *Hypnum* sp.	[74]
Strain F0358	Xylariaceae sp.	KU747599	Endophyte in *Phlebodium pseudoaureum*	[67]
Strain ATCC 58729	*Parahypoxylon papillatum*	NG_066379	from **epitype** of *Hypoxylon papillatum*	[68]

## 3. Results

### 3.1. Phylogeny

After DNA amplification, sequences for the ITS-5.8S region and the partial LSU D1/D2 region were obtained from 15 specimens. Four additional specimens yielded only the ITS-5.8S region, and one specimen, HUTPL(F)-1533, failed to amplify (Table 1).

**Figure 1:**
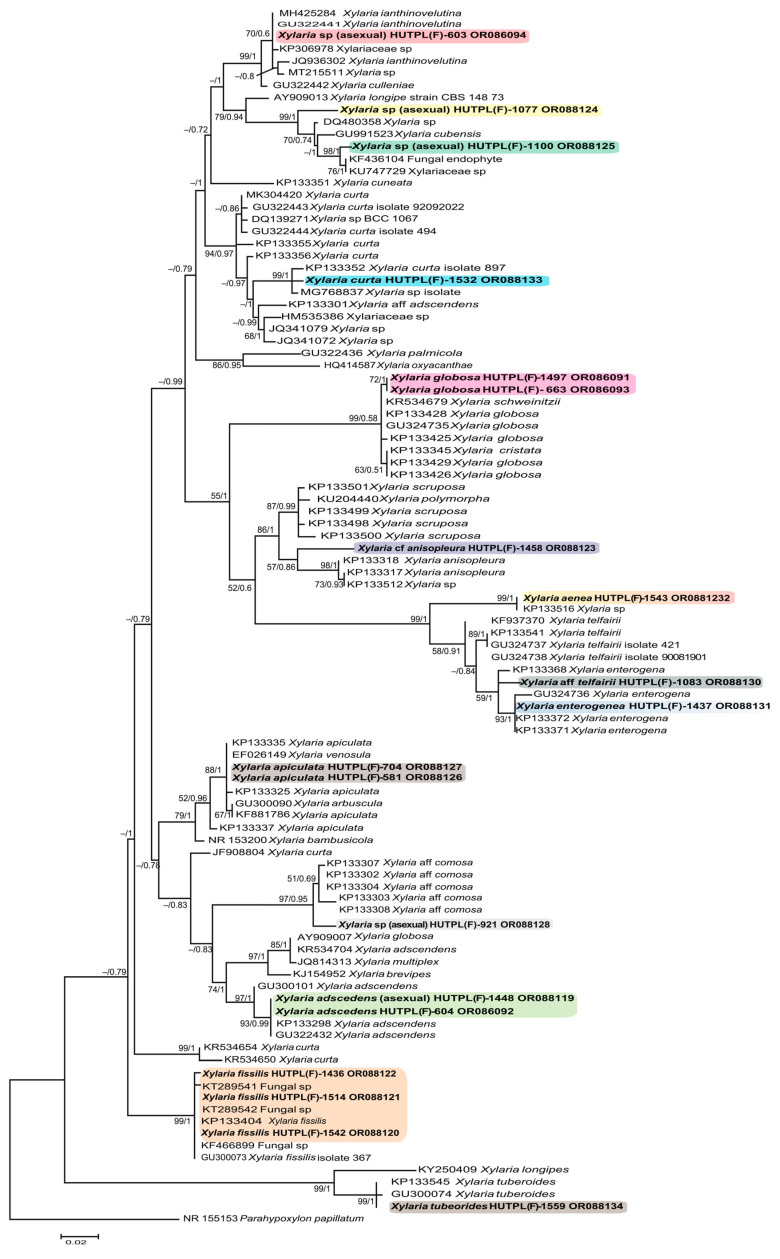
Phylogenetic tree ITS-5.8S regions with new sequences (highlighted in color) from *Xylaria* spp. from Southern Ecuador. Values at the nodes correspond to Maximum likelihood BS (left) and BPP (right), respectively. Only values > 50% and 0.5 are shown on nodes. Tree was rooted with *Parahypoxylon papillatum* (NR_155153).

The phylogenetic trees (Figure 1 and Figure S1) inferred using the ITS-5.8S and partial LSU DNA regions, showed partial agreement between some morphologically and phylogenetically defined species. This was evident in taxa like *Xylaria* cf. *anisopleura* and *Xylaria* aff. *telfairii* (Table 1; Figure 1 and Figure S1), which belong to species complexes with overlapping morphological traits [19].

The ITS-5.8S sequence for *Xylaria adscendens* showed high similarity to published sequences of the same species (GU322432; KP133298). However, the LSU sequence did not cluster with *X. adscendens* but instead grouped with a likely misidentified sequence of *Xylaria curta* (JF773598; Table 2, Figure S1), which was isolated as an endophyte from *Taxus globosa.*

The ITS-5.8S phylogenetic tree allowed the placement of sequences from several anamorphic specimens into species: HUTPL(F)-1448 as *Xylaria adscedens*; HUTPL(F)-704 as *Xylaria apiculata*; HUTPL(F)-1436 as *Xylaria fissilis* and HUTPL(F)-663 as *Xylaria globosa*. However, other sequences from anamorphic specimens were only closely related to species-level identifications [i.e., HUTPL(F)-603 related to *Xylaria cuneata* KP133351; HUTPL(F)-921 close together *Xylaria* aff. *comosa* KP133308; HUTPL(F)-1077 and HUTPL(F)-1100 related to *Xylaria cubensis* GU991523]. The remaining sequences from anamorphic specimens [HUTPL(F)-603, HUTPL(F)-921, HUTPL(F)-1077, HUTPL(F)-1100] clustered separately and are provisionally designated as *Xylaria* spp. Additionally, the sequence from the *Xylaria aenea* morphospecies did not cluster with any described species (Table 2, Figure 1 and Figure S1), supporting the notion that no reference sequences for this taxon are currently available.

### 3.2. Morphology

Among the 20 *Xylaria* collections analyzed, 11 teleomorph specimens were identified to species level using an integrative approach combining morphological and molecular analyses. These analyzed specimens corresponded to 10 species of *Xylaria*: *Xylaria adscendens*, *X. aenea*, *X.* cf. *anisopleura*, *X. apiculata*, *X. curta*, *X. enterogena*, *X. fissilis*, *X. globosa*, *X. aff. telfairii*, and *X. tuberoides*. Nine collections were found only in the anamorph state. Four [HUTPL(F)-1448, HUTPL(F)-604, HUTPL(F)-663, HUTPL(F)-1436] were assigned by molecular methods to species level (Table 1), and the other five [HUTPL(F)-603, HUTPL(F)-921, HUTPL(F)-1077, HUTPL(F)-1100, HUTPL(F)-1533] were not classified into any described species (Table 1).

### 3.3. Taxonomy

***Xylaria adscendens*** Fr., Linnaea 5: 537 (1830)

MycoBank No: 190364

Figure 2A–G

**Synonymy**: ≡*Xylaria adscendens* (Fr.) Fr., Nova Acta Regiae Societatis Scientiarum Upsaliensis 1: 128 (1851); *≡Xylosphaera adscendens* (Fr.) Dennis, Kew Bulletin 13 (1): 102 (1958); *≡Xylosphaera hypoxylon subsp. adscendens* (Fr.) Dennis, Bulletin du Jardin Botanique de l’État à Bruxelles 31: 124 (1961); *≡Xylaria hypoxylon subsp. adscendens* (Fr.) D. Hawksw., Transactions of the British Mycological Society 61 (1): 199 (1973); *Sensu* Mycobank Database [3].

**Description.** *Stromata* usually solitary and occasionally forming small groups, cylindrical and slightly apiculate without branches, externally black, rough to cracked, and the entostromata white to cream, 32–50 × 3–5 mm with stipe of 8–22 × 1–2 mm. In the immature asexual state, the stromata exhibit a slightly creamy white coloration at the upper part. *Perithecia* totally immersed into the stromata, black with ostioles slightly papillate. *Asci* unitunicate, inoperculate, and cylindrical with eight ascopores, 75–84 × 4–5 μm.

*Apical apparatus* amyloid, 2–3 × 1–2 μm, tubular, parallel, and slightly flattened at the upper apical part. *Ascospores* uniseriate, guttulate, with a straight spore-length germ slit [(10–) 11 (–12) × (4–) 4.5 (–5) μm], elliptical and inequilateral or oblique with Q value 2.4 μm, dark brown colored. *Paraphyses* absent.

**Examined specimens.** SOUTH AMERICA: ECUADOR. Zamora Chinchipe, el Tambo, Estación Científica San Francisco, path AT (1900 m.a.s.l.), growing on decaying wood, 19 May 2015, A. Chamba EM-166, HUTPL(F)-1448; same locality, path AT (1900 m.a.s.l.), growing on decaying wood, 3 April 2014, D. Cruz IR-109, HUTPL(F)-604 (asexual state only).

**Figure 2:**
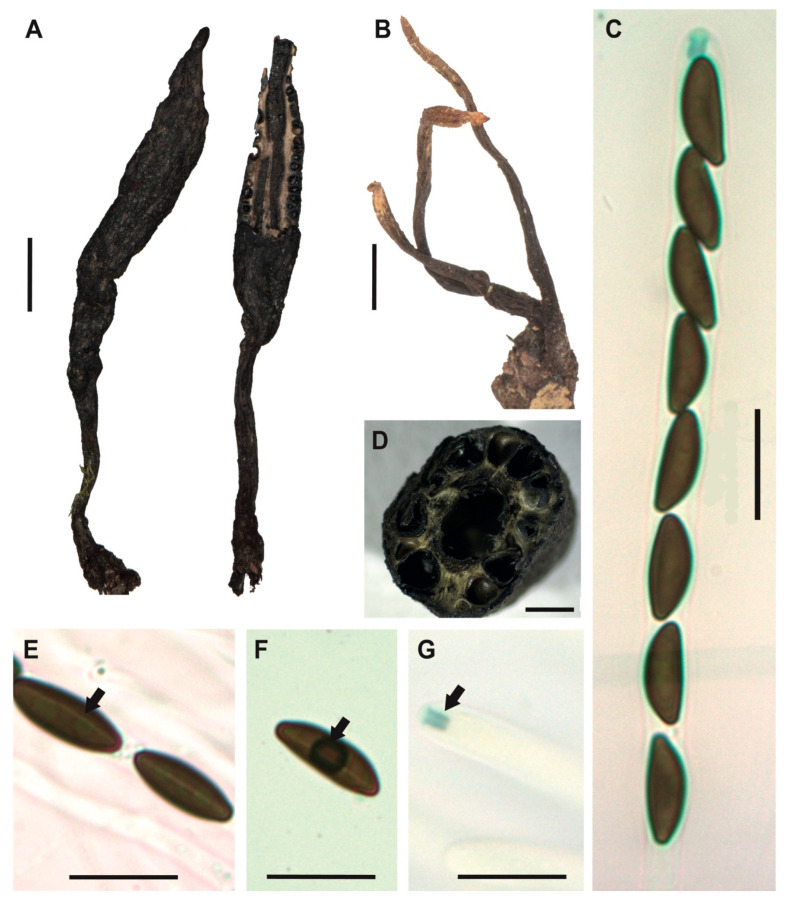
*Xylaria adscendens* [HUTPL(F)-1448]. (**A**) external macroscopic characteristics of the ascocarp and stipe. (**B**) Asexual state of *Xylaria adscendens* [HUTPL(F)-604]. (**C**) Ascus with eight ascospores (brown) and amyloid apical apparatus to Melzer’s reagent. (**D**) Transversal section showing the black perithecia around the stroma and a central hollow in the ascocarp. (**E**,**F**) Ascospores showing the germ slit and one guttulate (black arrow). (**G**) Amyloid apical apparatus at the tip of the ascus (black arrow). Scale bars: 1 cm (**A**); 1 cm (**B**); 1 mm (**C**); 10 μm (**D**–**G**).

**Remark.** Our specimen HUTPL(F)-1448 (Figure 2), characterized by ellipsoid and inequilateral ascospores, exhibits a general morphological resemblance to several specimens identified as *Xylaria adscendens*. Notably, it aligns with descriptions from tropical South American forests, such as Brazilian collections with ascospores dimensions of 9–14 × 4–5 μm [75] or (9–)11–14.5(–15) × 3–5 μm [30]. Comparable measurements have also been reported from cloud forests in Mexico for ascospores of *X. adscendens* with (9–) 10.5–13(–14) × 4.5–5 μm [76], and Papua Nueva Guinea 11–14 × 4.5 μm [77,78].

The specimen HUTPL(F)-1448 described here is consistent with *Xylaria adscendens*, differing from the branched stromata of *X. hypoxylon* [75] and the massive, fused stromata of *X. multiplex* [76,77], while sharing the pointed apex and long, striped peeling layer characteristic of the *X. hypoxylon* aggregate [20]. Based on its morphology and phylogenetic placement with other ITS-5.8S sequences identified as *X. adscendens* from ascomata-derived strains, we retain the identification of specimen HUTPL(F)-1448 and HUTPL(F)-604 as *Xylaria adscendens*.

***Xylaria aenea*** Mont., Annales des Sciences Naturelles Botanique 3: 100 (1855).

MycoBank No: 535638

Figure 3A–H

**Synonymy:** ≡*Xylaria aenea* Mont. (1855); ≡*Xylosphaera aenea* (Mont.) Dennis, Kew Bulletin 13 (1): 102 (1958); *Sensu* Mycobank Database (http://www.mycobank.org/).

**Description.** *Stromata* solitary, unbranched, clavate and rounded at the top, with fluted or large wrinkled black surface, but white to cream internally, 99 × 8 mm with stipe of 47 × 4 mm. *Perithecia* totally immersed in the stromata, black with discoid ostioles slightly papillate. *Asci* with eight partially biseriate ascospores 133–169 × 5–11.5 μm. *Apical apparatus* amyloid to Melzer’s reaction, 4–5 × 3–4 μm, tubular, parallel, and slightly urn-shaped, with a narrow neck and a broad opening. *Ascospores* biseriate, with two to three internal oil drops, partial ventral germ slit [(30–)33.5(–37) × (5–)6(–7) μm], elliptical, inequilateral or partially falcate with Q value 5.6, and dark brown color. *Paraphyses* absent.

**Examined specimens.** SOUTH AMERICA: ECUADOR. Zamora Chinchipe, el Tambo, Estación Científica San Francisco, path T2 (2500 m.a.s.l.), growing on decaying wood, 25 August 2015, D. Cruz EM-593, HUTPL(F)-1543.

**Remark.**

**Figure 3:**
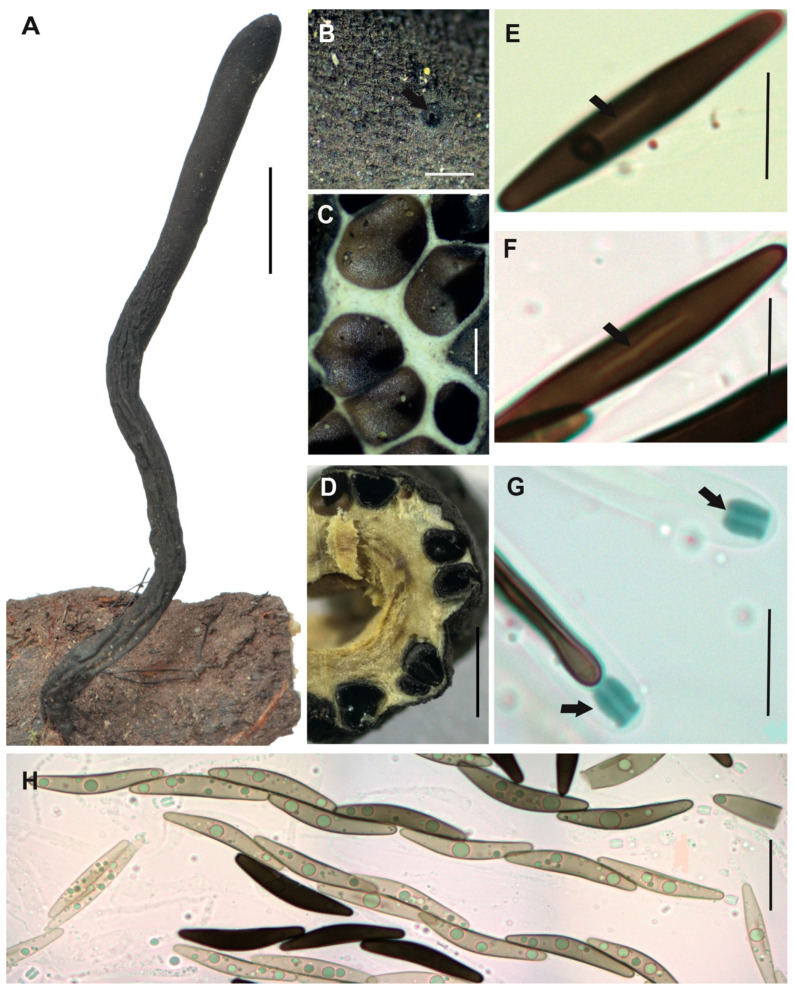
*Xylaria aenea* [HUTPL(F)-1543]. (**A**) External macroscopic characteristics of the ascocarp. (**B**) Discoid open ostiole (black arrow). (**C**,**D**) Longitudinal and transversal section showing the brown to black perithecia totally immersed within stroma. (**E**,**F**) Ascospores showing the germ slit (black arrows). (**G**) Amyloid apical apparatus (black arrows) at the asci tip. (**H**) Asci with young ascospores with central guttule and biseriate arrangement (preparation in KOH). Scale bars: 2 cm (**A**); 0.4 mm (**B**); 0.5 mm (**C**); 1 mm (**D**); 10 μm (**E**–**G**); 20 μm (**H**).

Our specimen HUTPL(F)-1543 (Figure 3) matches with *Xylaria* cf. *aenea* as interpreted by Rogers [31] and epitypified by Ju and Hsieh [79], particularly in the shape and size range of both stromata “cylindrical or clavate, unbranched, rounded and fertile at apex, on a short to long stipe, up to 7 cm in total length × 0.4–2.1 cm broad, 2.5–5.8 cm long at fertile parts, 0.7–5.0 cm long at stipes; surface lacking perithecial mounds” and ascospores “(26.5–)28.5–34(–37.5) × (4.5–)5–6(–8) µm brown to dark brown, unicellular, falcate, with broadly rounded ends, bearing a tiny cellular appendage on one end”. Rogers [31] examined and compared specimens originally described from Venezuela by Dennis [80], subsequently, Ju and Hsieh [79] studied the same material examined by Dennis and proposed an epitypification for *Xylaria aenea* (VENEZUELA. Aragua, Colonia Tovar, Fendler A. 253, as *X. aenea* by Dennis, R.W.G. [K(M) 169675 ex Berk. herb.] EPITYPE). The concept of *Xylaria aenea* based on the epitype aligns with our specimen in terms of ascospore shape and dimensions. However, it clearly differs in stromatal morphology, presenting shorter, more compact stromata with a finely cracked, bronze-colored surface. In contrast, our specimen develops larger stromata, reaching up to 99 mm in length and 8 mm in width, with a prominently fluted or wrinkled surface, whereas the epitype measures only 70 mm in length and 4–21 mm in width.

Based on the strong morphological correspondence in ascospore characteristics, we assign our specimen HUTPL(F)-1543 to *Xylaria aenea*. The observed differences in stromatal size and surface texture are interpreted as intraspecific variation, likely influenced by environmental conditions.

To date, no reference sequences for *Xylaria aenea* exist in GenBank or other databases. However, the ITS-5.8S sequence of our specimen matches the unidentified “*Xylaria* sp.” reported by Thomas [33], and shows close similarity to sequences of *X. enterogenea*, suggesting a possible genetic relationship.

***Xylaria*** **cf.** ***anisopleura*** (Mont.) Fr., Sylloge generum specierumque plantarum cryptogamarum: 204 (1851)

MycoBank No: 190626

Figure 4A–F

**Synonymy:** ≡*Hypoxylon anisopleuron* Mont., Annales des Sciences Naturelles Botanique 13: 348 (1840); ≡*Xylosphaera anisopleura* (Mont.) Dennis, Kew Bulletin 13 (1): 102 (1958); *Sensu* Mycobank Database (http://www.mycobank.org/).

**Description**. *Stromata* gregarious, unbranched, subglobose to clavate, rounded and moriform surface to the top, commonly black to strong brown, with white entostromata limited at the stromata base by one black line, 9–12 × 3–4 mm with stipe of 2–4 × 0.7–1 mm. *Perithecia* totally immersed into the stromata, black with umbilicated ostioles. *Asci* cylindrical with eight ascospores, 142–164 × 9–12 μm. *Apical apparatus* amyloid to Melzer’s reaction, 4–5 × 3–4 μm, tubular slightly curved, parallel, with flattened at the upper apical part. *Ascospores* uniseriate, with one or two internal oil drops, partial spiral to oblique ventral germ slit [(19–)23(–28) × (7–)9(–13) μm], elliptical, inequilateral with Q value 2.5, and dark brown color. *Paraphyses* absent.

**Examined specimens.** SOUTH AMERICA: ECUADOR. Zamora Chinchipe, el Tambo, Estación Científica San Francisco, path T2 (2500 m.a.s.l.), growing on decaying wood, 02 June 2015, A. Chamba EM-506, HUTPL(F)-1458.

**Figure 4:**
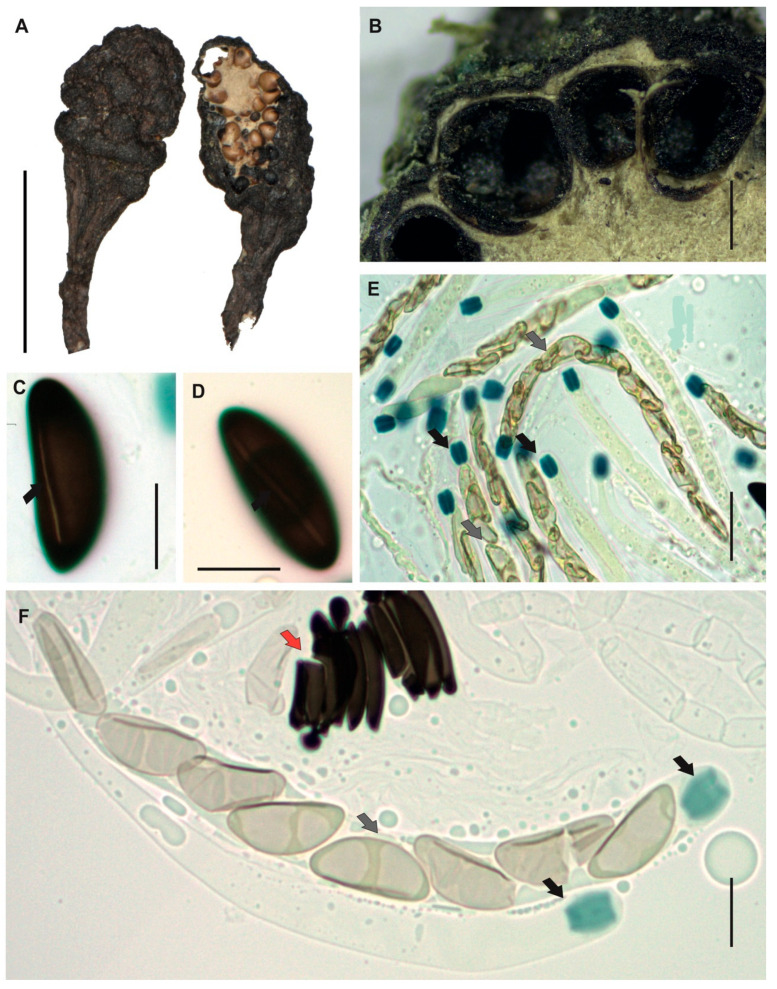
*Xylaria* cf. *anisopleura* [HUTPL(F)-1458]. (**A**) External macroscopic characteristics of the ascocarp. (**B**) Longitudinal and transversal section showing the black perithecia totally immersed into the stroma. (**C**,**D**) Ascospores, partial spiral to oblique ventral germ slit pore (black arrows). (**E**,**F**) Immature asci with uniseriate immature ascospores (grey arrows) with amyloid apical apparatus stained in blue at the asci tip (black arrows) with Melzer’s reagent, and mature collapsed ascospores (red arrow). Scale bars: 1 cm (**A**); 1 mm (**B**); 10 μm (**C**,**D**); 20 μm (**E**,**F**).

**Remark.** The specimen HUTPL(F)-1458 (Figure 4) with small and their partial spiral to oblique ventral germ slit in the ascospores correspond to *Xylaria anisopleura* applying the taxonomic keys [30,81]. Additionally, our specimen is morphologically like many descriptions of *X. anisopleura* from different tropical forest [30,75,78,81,82,83]. Our *X.* cf. *anisopleura* (HUTPL(F)-1458) is different to the specimen HUTPL(F)-1497 presented here as *Xylaria globosa*, specially by the shorter stromata and the partial spiral to oblique ventral germ slit.

However, Rogers [31] considered to *X. anisopleura* part of a complex group closely related to morphospecies such as *X. globosa*, which was later treated as synonym by Van der Gucht [75] and Hamme and Trinidad [29], as well as to *X. polymorpha* and *X. scruposa* according to Dennis [80] and Cruz [84]. Due to the high degree of morphological overlap between *X. anisopleura* and *X. globosa*, Fournier [85] also treated both taxa as synonymous.

However, Dennis [80] suggests *Xylaria anisopleura* differs from *X. polymorpha* mainly by its smaller stromata, and Cruz [84] suggest it differs from *X. scruposa* by its larger ascospores [13–21 × 4.5–7 µm in *X. scruposa*], and in its distinctly moriform stromata.

***Xylaria apiculata*** Cooke, Grevillea 8 (46): 66 (1879)

MycoBank No: 190405

Figure 5A–G

**Synonymy:** ≡*Xylosphaera apiculata* (Cooke) Dennis, Kew Bulletin 13 (1): 102 (1958); *Sensu* Mycobank Database (http://www.mycobank.org/).

**Description.** *Stromata* grouped or solitary, unbranched, cylindrical, smooth when young and becoming vertically striping so called “zebra-stripping” coming splits with perithecial swelling, blackish color, with a sterile apex (without perithecia), and white to cream entostromata, 11–23 × 2–3 mm with stipe of 5–10 × 0.4–1 mm, slightly tomentose. *Perithecia* totally immersed into the stromata, present in the whole fertile area, black with umbilicated barely visible ostioles. *Asci* stipitate at the base and continue cylindrical with eight ascospores, 90–110 × 6–10 μm. *Apical apparatus* amyloid to Melzer’s reaction, 9–11 × 5–8 μm, tubular, parallel, and slightly flattened at the upper apical part. *Ascospores* uniseriate, with one or two internal oil drops, partial ventral germ slit [(14–)16(–18) × (6–)7(–8) μm], elliptical, inequilateral with Q value 2.3, and light brown to dark brown color. *Paraphyses* absent.

**Examined specimens.** SOUTH AMERICA: ECUADOR. Zamora Chinchipe, el Tambo, Estación Científica San Francisco, path AT (1900 m.a.s.l.), growing on decaying wood, 3 April 2014, A. Chamba IR-85, HUTPL(F)-581; same locality, path AT (1900 m.a.s.l.), growing on decaying wood, 3 July 2014, A. Chamba IR-211, HUTPL(F)-704.

**Remark.** *Xylaria apiculata* is distinguished by short stromata less than 3 mm and surface texture reassembling “zebra-stripping” typically in this species, as well as ascospores measurements. The ascospores of *X. apiculata* have been reported by other authors as measuring 16–18.5(19) × (4)5–6.5 μm [75] and 16–21 × 6–7.5 μm [80]. This species differs from closely related taxa such as *Xylaria venosula*, which has significantly larger ascospores measuring 21–25 × 7.5–8 μm [80] and has been considered a synonym of *Xylaria xylaroides* by Hladki and Romero [26].

Phylogenetically, the ITS-5.8S sequences from specimens HUTPL(F)-581 and HUTPL(F)-704 clustered with sequences identified as *X. apiculata* from the Ecuadorian cloud forest [33]. Interestingly, one sequence labeled as *X. venosula* also grouped within this clade, possibly due to misidentification, as the two species are morphologically distinct.

Additionally, LSU data show that our *X. apiculata* specimens are closely related to *X. arbuscula*. However, key morphological differences—particularly in stromatal architecture and ascospore dimensions support their distinction as separate species (see *X. arbuscula* description).

***Xylaria curta*** Fr., Nova Acta Regiae Societatis Scientiarum Upsaliensis 1: 126 (1851)

MycoBank No: 179336

Figure 6A–G

**Synonymy:** ≡*Xylosphaera curta* (Fr.) Dennis, Kew Bulletin 13 (1): 103 (1958); *Sensu* Mycobank Database (http://www.mycobank.org/).

**Description.** *Stromata* 13–24 × 3–6 mm, with stipe of 8–11 × 1–3 mm, clustered in groups of two to six fruit bodies, cylindrical, rough, rounded to the apex, blackish in color, but sometimes with external scales conferring brownish color; white to cream entostromata. *Perithecia* globose, totally immersed in the stromata, black with papillate ostioles. *Asci* cylindrical with eight ascospores, 65–77 × 4–4.8 μm. *Apical apparatus* amyloid to Melzer’s reaction, 2–3 × 1–2 μm, tubular, parallel, and narrow at the base, becoming slightly flattened toward the apical portion. *Ascospores* uniseriate, guttulate central, with ventral straight germ slit commonly covering the whole spore (9–)10(–11) × (4–)4.5(–5) μm, elliptical, inequilateral with Q value 2.2, and brown color. *Paraphyses* absent.

**Examined specimens.** SOUTH AMERICA: ECUADOR. Zamora Chinchipe, el Tambo, Estación Científica San Francisco, path T2 (2500 m.a.s.l.), growing on decaying wood, 25 August 2016, D. Cruz EM-582, HUTPL(F)-1532.

**Figure 6:**
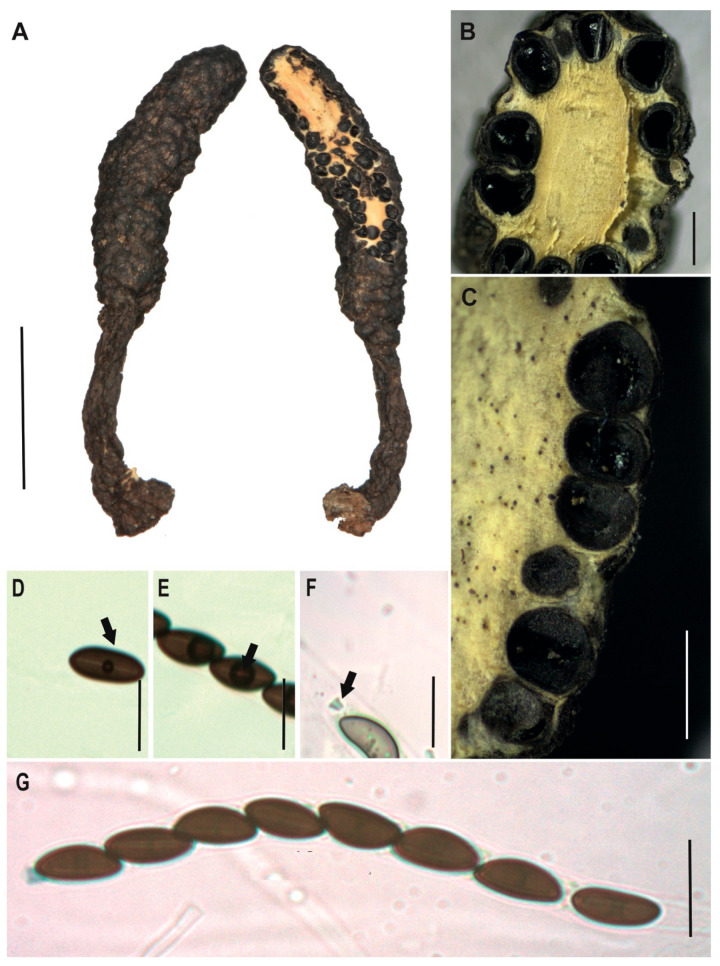
*Xylaria curta* [HUTPL(F)-1532]. (**A**) Macroscopic characteristics of the claviform ascocarp. (**B**,**C**) Longitudinal and transversal section showing the black and swollen perithecia immersed into the stroma. (**D**,**E**) Ascospores, with ventral straight germ slit (black arrows). (**F**) amyloid apical apparatus (black arrow) at the immature ascus tip. (**G**) Ascus with eight ascospores stained with Melzer’s reagent producing amyloid apical apparatus (blue). Scale bars: 1 cm (**A**); 1 mm (**B**,**C**); 10 μm (**D**–**G**).

**Remark.** The specimen [HUTPL(F)-1532] (Figure 6), based on its morphology, fits within the species *Xylaria curta* according to the taxonomic key provided by Dennis [80]. However, the size of the stroma appears to be highly variable (ranging from 1.8 cm to 5 cm), as also reported in specimens of *X. curta* from Mexico, Brazil, Costa Rica, France, and Venezuela [30,75,83].

Hsieh [20] placed *Xylaria curta* within the *X. corniformis* aggregate, which also includes *X. feejeensis*, *X. montagnei*, and *X. plebeja*, all characterized by similar stromatal textures finely cracked or wrinkled and small, ellipsoid ascospores. Our specimen of *X. curta* resembles *X. feejeensis* in ascospore size (8–12 × 4–5 µm), but differs by having stouter, often sessile and clustered stromata, with a white to cream immature surface that is distinctly cracked into persistent angular or rounded scales between the ostiolar papillae.

Phylogenetically, the ITS-5.8S and LSU sequences of specimen HUTPL(F)-1532 cluster with other *X. curta* sequences (AB376702, GU322444, KP133352, KP133356) derived from axenic cultures of ascomata, as reported by Thomas [33], Hsieh [20], and Okane [71]. These analyses indicate that *X. curta* forms a distinct clade, separate from closely related taxa, supporting its recognition as an independent species within the complex. Morphologically, our specimen differs from *X. montagnei* and *X. feejeensis* by its shorter stromata, and from *X. plebeja* by having broader ascospores.

***Xylaria enterogena*** Mont., *Syll. gen. sp. crypt.* (Paris): 203 (1856)

MycoBank No: 250347

Figure 7A–E

**Figure 7:**
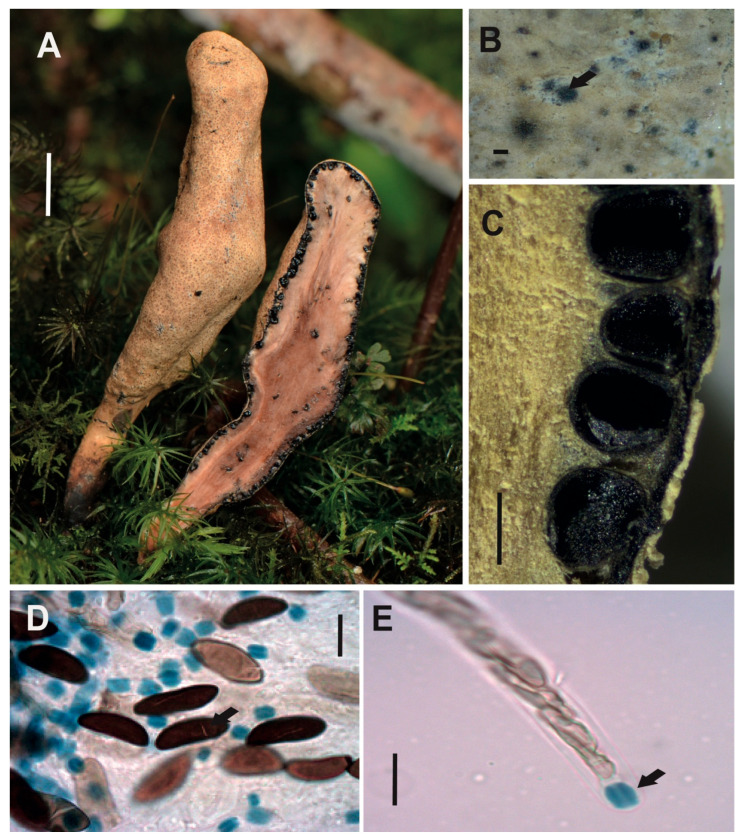
*Xylaria enterogenea* [HUTPL(F)-1437]. (**A**) External macroscopic characteristics of fresh ascocarp. (**B**) Ostiolar surfaced black ring (black arrow). (**C**) Longitudinal section showing black and swollen perithecia immersed into the stroma. (**D**) Ascospores with partial ventral germ slit slightly oblique to one apex (black arrow), and amyloid apical apparatus (blue). (**E**) Amyloid apical apparatus (black arrow) at the immature ascus tip. Scale bars: 1 cm (**A**); 0.5 mm (**B**); 0.6 mm (**C**); 10 μm (**D**,**E**).

**Description.** *Stromata* solitary, unbranched, clavate with rounded apex, yellowish cream in color, 63 × 4–6 mm with stipe 16 × 3 mm, with a smooth and hard surface; entostromata wet and white when fresh, darkening and becoming hollow in age. *Perithecia* immersed into the stromata, with ostiolar black rings. *Asci* cylindrical with eight ascospores, 131–142 × 6–11 μm. *Apical apparatus* amyloid to Melzer’s reaction, 5–6 × 3–5 μm, tubular slightly curved, parallel, with flattened at the upper apical part. *Ascospores* uniseriate, with one or two guttules, partial ventral germ slit sometimes oblique to one apex (18–)21(–24) × (5–)7(–9) μm, elliptical, inequilateral with Q value 3, and brown color. *Paraphyses* absent (Figure 7).

**Examined specimens.** SOUTH AMERICA: ECUADOR. Zamora Chinchipe, el Tambo, Estación Científica San Francisco, path T2 (2500 m.a.s.l.), growing on decaying wood, 19 May 2016, A. Chamba EM-485, HUTPL(F)-1437.

**Remark.** *Xylaria enterogena* is usually confusing with *Xylaria telfairii* (Berk.) Sacc., specially by the ascospores shape and measurements. Rogers et al. (1988) [31] report to *X. enterogena* with ascospores (13–)17.5–25 × 6–7.5 μm and *X. telfairii* with ascospores (15–)16–21 × 6–7.5 μm. Based on similar ascospore dimensions, these species were suggested as conspecific by Dennis (1956) [80], who considered that *X. enterogena* to be a young state of *X. telfairii*.

The specimen HUTPL(F)-1437 (Figure 7) has characteristics close to the description of *X.* aff*. enterogena* (*sensu* [81]) from Mexico, which has 68 mm stromata length and ascospores measurements (19–)20–23(–25) × 8–9.5(–10). Here, the specimen HUTPL(F)-1437 differ to the specimen HUTPL(F)-1083 for the stromata 95 × 6–14 mm (fertile portion 38 mm length), with long brown, and smooth stipe 57 × 4 mm, but similar ascospores shape and measurements (18–)21(–25) × (5–)7(–9) μm, and molecular placement (Figure 7). Molecular data show a close relationship between our specimen HUTPL(F)-1437 *Xylaria enterogenea* and *X. telfairii*. This affinity is further supported by recent analyses by Forin [19], which include the sequence [HUTPL(F)-1437, OR088131] within a distinct clade referred to as the *Xylaria enterogenea* complex, positioned close to but separate from the *X. telfairii* clade.

***Xylaria fissilis*** Ces. Atti dell’Accademia di Scienze Fisiche e Matematiche Napoli 8 (3): 16 (1879)

MycoBank No: 145665

Figure 8A–G

**Description.** *Stromata* gregarious mostly with two lateral branches, cylindrical, black color, slightly cracked, flattened flabelliform, pointing towards the apex, with blackish entostromata, internal 17–18 × 1–4 mm, with stipe 3.5–11 × 1–2.5 mm. *Perithecia* mostly immersed into the stromata, black with papillate ostioles. *Asci* cylindrical with eight ascospores, 66–96 × 4–6 μm. *Apical apparatus* amyloid to Melzer’s reaction, 2–4 × 2–3 μm, tubular, parallel, and slightly flattened at the upper apical part. *Ascospores* uniseriate, with germ slit, almost full spore length (10–)13(–14) × 4(–6) μm, navicular to elliptical, inequilateral with Q value 3.2 and brown color. *Paraphyses* absent.

**Examined specimens.** SOUTH AMERICA: ECUADOR. Zamora Chinchipe, el Tambo, Estación Científica San Francisco, path AT (1900 m.a.s.l.), growing on decaying wood, 21 July 2016, A. Chamba EM-564, HUTPL(F)-1514; same locality, path T2 (2500 m.a.s.l.), growing on decaying wood, 25 August 2015, D. Cruz EM-592, HUTPL(F)-1542; same locality, path AT (1900 m.a.s.l.), growing on decaying wood, 19 May 2015, A. Chamba EM-484, HUTPL(F)-1436.

**Remark.** When follow the taxonomic key given by Hladki and Romero [24,26] for *Xylaria* spp. from Argentina our description for the specimens revised here fit into the *Xylaria fissilis* specially by the ascospores measurements 14.5–17.5 × 4–7 μm. Trierveiler Pereira [30] mention that *X. nigromedullosa* presents similar dark brown to black entostromata as described for *X. fissilis* and *X. luxurians* (Rehm) Lloyd. However, our specimens, identified as *Xylaria fissilis*, differs from *X. luxurians* Dennis [80] and *X. nigromedullosa* Trierveiler Pereira [30] in their stromatic features which are slightly cracked, flattened, and flabelliform with an apex-oriented growth as well as in ascospore size 21–24 × 8–9 and 7–9.5 × 4–5 µm respectively and the presence of a germ slit extending nearly the entire length of the ascospores.

***Xylaria globosa*** (Spreng.) Mont. (1855)

MycoBank No: 292028

Figure 9A–G

**Synonymy:** ≡*Hypoxylon globosum* (Spreng. ex Fr.) Fr., Nova Acta Regiae Societatis Scientiarum Upsaliensis 1: 130 (1851); *Sensu* Mycobank Database (http://www.mycobank.org/).

**Description.** *Stromata* usually solitary or sometimes grouped, cylindrical to obclavate, rounded to the apex, blackish color, roughly and slightly cracked, with white to cream entostromata, 16–29 × 4–8 mm with stipe sometimes difficult to distinguish from the stromata 5–14 × 1–2 mm. In asexual state present red exudates on young stromata. *Perithecia* totally immersed into the stromata, black with papillate ostioles. *Asci* cylindrical with eight ascospores, 146–210 × 8–14 μm. *Apical apparatus* amyloid to Melzer’s reaction, 7–10 × 5–7 μm, tubular slightly curved, parallel, with flattened at the upper apical part. *Ascospores* uniseriate, guttulate commonly with one to two oil drops, with partial spiral ventral germ slit [(25–)28(–33) × (8–)9(–11) μm], elliptical, inequilateral with Q value 3, and strong brown color. *Paraphyses* absent.

***Examined specimens*****.** SOUTH AMERICA: ECUADOR. Zamora Chinchipe, el Tambo, Estación Científica San Francisco, path T2 (2500 m.a.s.l.), growing on decaying wood, 7 July 2016, A. Chamba EM-545, HUTPL(F)-1497; same locality, path AT (1900 m.a.s.l.), growing on decaying wood, 5 June 2014, A. Chamba IR-170, HUTPL(F)-663 (asexual state).

**Figure 9:**
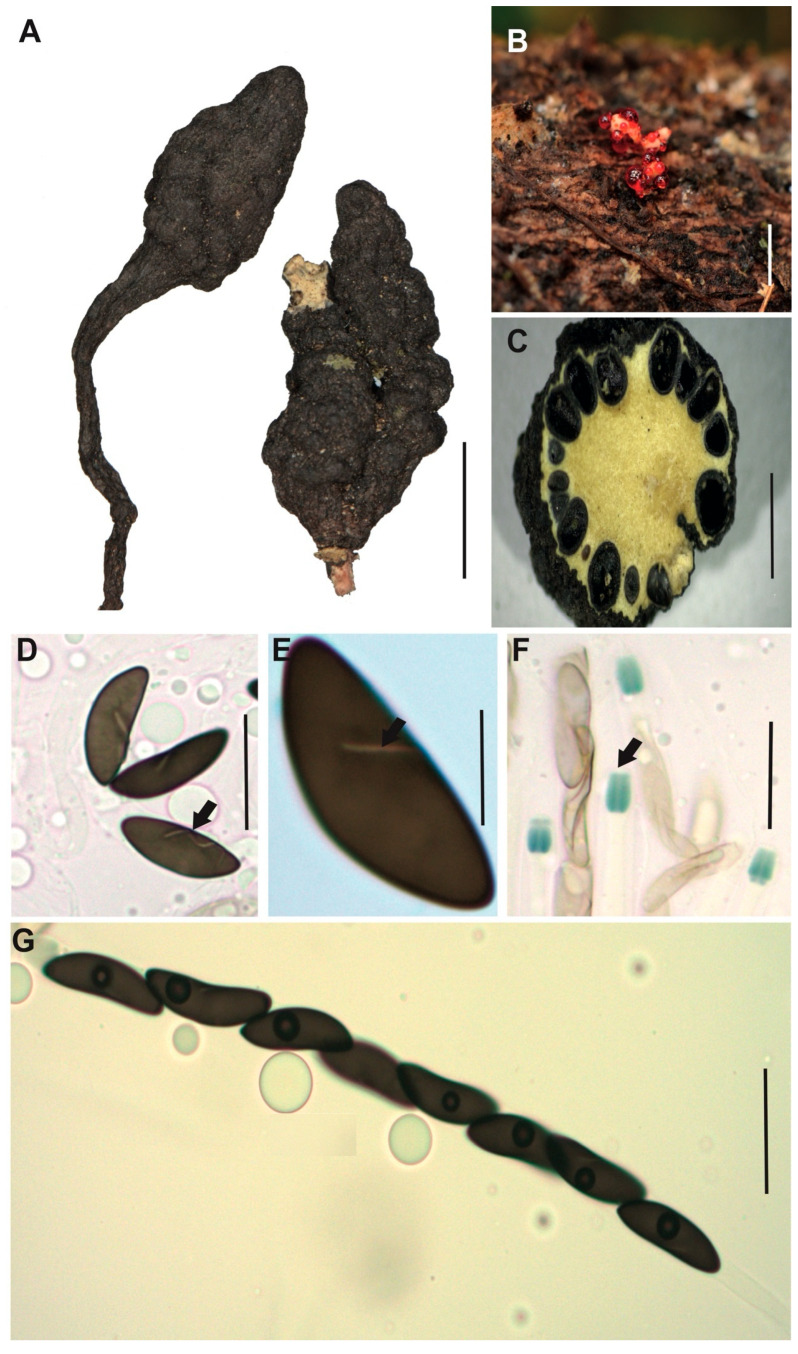
*Xylaria globosa* [HUTPL(F)-1497]. (**A**) External macroscopic characteristics of the ascocarp. (**B**) Asexual state with red exudates on young stromata [HUTPL(F)-663]. (**C**) Transversal section showing black and swollen perithecia immersed into the stroma. (**D**,**E**) Ascospores, with partial spiral ventral germ slit (black arrow). (**F**) Amyloid apical apparatus (black arrow) at the immature asci tip. (**G**) Ascus with eight ascospores in Melzer’s reagent. Scale bars: 1 cm (**A**); 2 cm (**B**); 2 mm (**C**); 10 μm (**D**–**F**); 20 μm (**G**).

**Remark.** *Xylaria globosa* is distinguished by its large elliptical ascospores, as observed in specimen HUTPL(F)-1497 (Figure 9). This specimen fit well within the concept of *X. globosa* when applying the taxonomic key for *Xylaria* species from Argentina proposed by Hladki and Romero [26] specially by the ascospores size 22–30 × 8–9.5 μm. The other specimen HUTPL(F)-663 is determined as *X. globosa* because their asexual state presents a red exudate, a morphological character typical for this species (Figure S1).

Phylogenetically, our ITS-5.8S sequences cluster with reference sequences of *Xylaria globosa* (e.g., GU324735, KP133429), forming a well-supported clade consistent with that reported by Forin [19], and clearly separated from the clade containing *Xylaria* cf. *anisopleura* [HUTPL(F)-1458]. Although *X. globosa* has been previously synonymized with *X. anisopleura* due to morphological variability, the ascospores of our specimens [(25–)28(–33) × (8–)9(–11) μm] closely match the measurements reported by Hladki and Romero [26], supporting their identification as *X. globosa*. Furthermore, Forin [19] identified *Xylaria aurantiorubroguttata* as the sister group to *X. globosa*, but distinguishable by morphological traits such as slightly shorter ascospores (~23.9 × 8 μm) with a non-sigmoid, oblique germ slit.

***Xylaria*** **aff.** ***telfairii*** (Berk.) Sacc., Sylloge Fungorum 1: 320 (1882)

MycoBank No: 191825

Figure 10A–F

**Synonymy:** ≡*Sphaeria telfairii* Berk., Annals and Magazine of Natural History 3: 397 (1839) [MB#165107] ≡*Xylosphaera telfairii* (Berk.) Dennis, Kew Bulletin 13 (1): 106 (1958); *Sensu* Mycobank Database (http://www.mycobank.org/).

**Description.** *Stromata* solitary unbranched, cylindrical to slightly clavate, and rounded apex, cream to brownish, smooth and hard surface, with cream entostromata, without ventral hole, 95 × 6–14 mm (fertile portion 38 mm length), with long black smooth stipe 57 × 4 mm. *Perithecia* immersed into the stroma, black with umbilicate ostioles. *Asci* cylindrical and thin at the basal part with eight ascospores, (125–)142–153 × 6–11 μm. *Apical apparatus* amyloid to Melzer’s reaction, 5–6 × 3–5 μm, tubular slightly curved, parallel, with flattened at the upper apical part. *Ascospores* uniseriate, with one or two internal oil drops, partial ventral germ slit sometimes oblique to one apex (18–)21(–25) × (6–)7(–9) μm, elliptical, inequilateral with Q value 3, and brown color. *Paraphyses* absent.

***Examined specimens*****.** SOUTH AMERICA: ECUADOR. Zamora Chinchipe, el Tambo, Estación Científica of San Francisco, path T2 (2500 m.a.s.l.), growing on decaying wood, 05 March 2015, A. Chamba EM-166, HUTPL(F)-1083.

**Figure 10:**
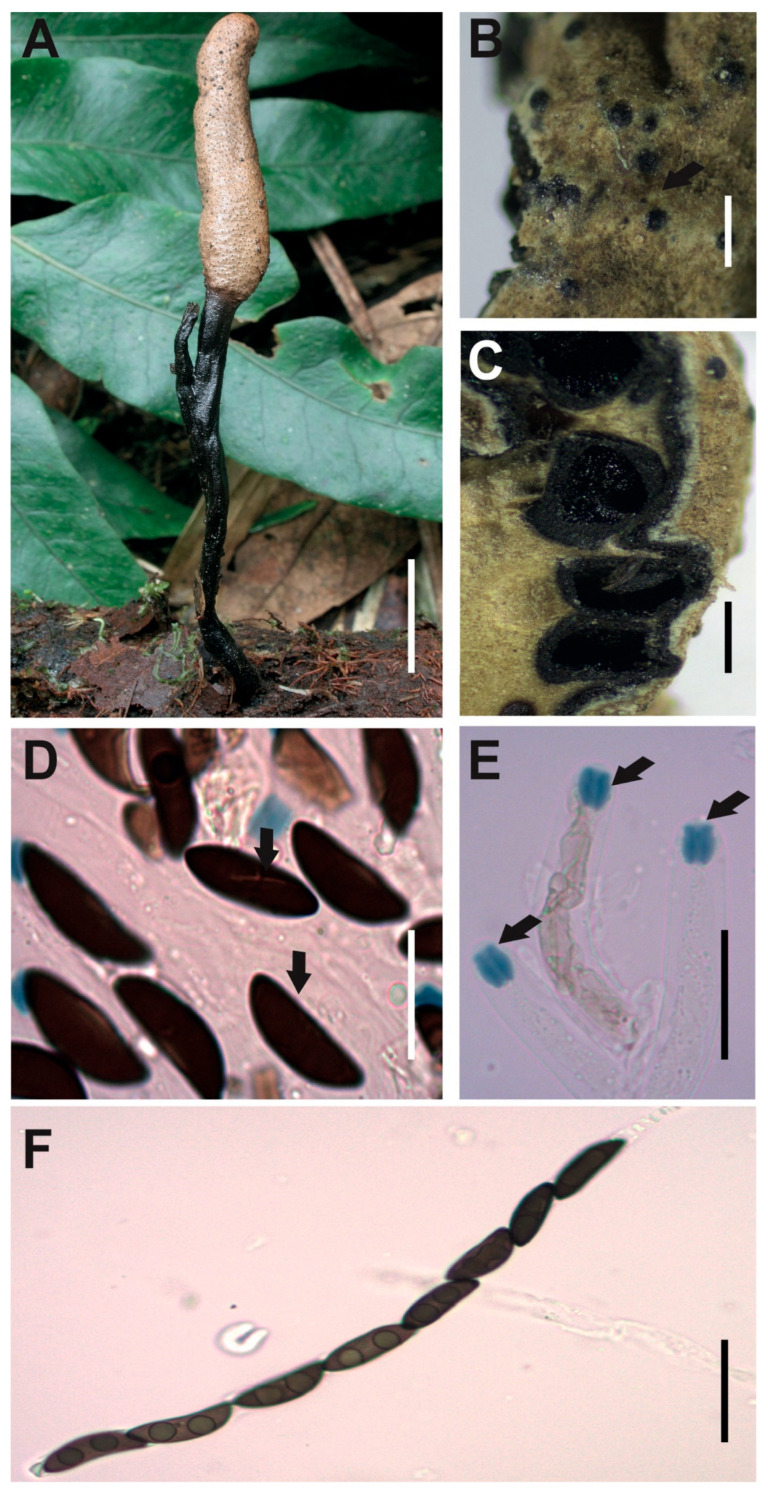
*Xylaria* aff. *telfairii*. (**A**) External macroscopic characteristics of fresh ascocarp. (**B**) Ostiolar surface points (black arrow). (**C**) Transversal section showing black and swollen perithecia immersed into the stroma. (**D**) Ascospores with partial ventral germ slit slightly oblique to one apex (black arrow), and amyloid apical apparatus (blue). (**E**) Amyloid apical apparatus (black arrow) at the immature asci tip. (**F**) Ascus with eight ascospores guttulate (brown) in KOH. Scale bars: 2 cm (**A**); 0.5 mm (**B**); 0.4 mm (**C**); 10 μm (**D**,**E**), 20 μm (**F**).

**Remark.** Morphologically, our specimen HUTPL(F)-1083 is closely related to *Xylaria enterogena* and *X. telfairii* by having cream to brownish stromata (Figure 10), and similar shape and measurements of ascospores. The ascospores measurements (18–)21(–25) × (5–)7(–9) μm in the specimen HUTPL(F)-1083 are into the range of the ascospores measurements found in *X. enterogena* (15–)16–21 × 6–7.5 μm [31], *X. telfairii* (13–)17.5–25 × 6–7.5 μm [31], and *X. telfairii* (15–)16–22(–25) × 6–8 μm [80]. However, the entostromata in the specimen HUTPL(F)-1083 was not hollow, probably due to the perithecia maturity state [80].

The specimens HUTPL(F)-1083 and the specimen HUTPL(F)-1437 are different in color, size (fertile portion 38 mm and stipe 57 mm length in HUTPL(F)-1083, against 49 mm and 16 mm in HUTPL(F)-1437 respectively), and shape of the stroma, but identical in shape of the ascospores (Figure 10).

Based on morphology (Figure 10) the specimen HUTPL(F)-1083 is considered similar most of the microscopical shapes and measurements of *Xylaria telfairii* available in Dennis [80], and Rogers [31].

The morphological and phylogenetic evidence is not conclusive to confidently identify the specimen HUTPL(F)-1083 as *Xylaria telfairii*. Therefore, we refer to it as *Xylaria aff. telfairii*. Although Forin [19] includes our sequence within the *X. telfairii* clade, it also shows a close relationship to the *X. enterogenea* clade, highlighting the need for further analysis.

***Xylaria tuberoides*** Rehm, Hedwigia 40: 146 (1901)

MycoBank No: 181321

Figure 11A–G

**Description.** *Stromata* solitary, globose to subglobose, black, and hard surface, with white to cream entostromata, 12–16 × 8–11 mm with stipe 3–4 × 2–4 mm. *Perithecia* immersed into the stromata, black with slightly papillate ostioles. *Asci* cylindrical with eight ascospores, 156–203 × 7–12 μm. *Apical apparatus* amyloid to Melzer’s reaction, 5–6 × 3–5 μm, tubular, parallel, and slightly urn-shaped, with a narrow neck and a broad opening. *Ascospores* uniseriate, with two or three internal oil drops, partial ventral germ slit slightly clear (24–)28(–32) × (6–)7(–11) μm, elliptical, inequilateral with Q value 3.9, and brown color. *Paraphyses* absent.

**Examined specimens.** SOUTH AMERICA: ECUADOR. Zamora Chinchipe, el Tambo, Estación Científica San Francisco, path T2 (2500 m.a.s.l.), growing on decaying wood, 3 September 2015, J.S. Eguiguren EM-609, HUTPL(F)-1559.

**Figure 11:**
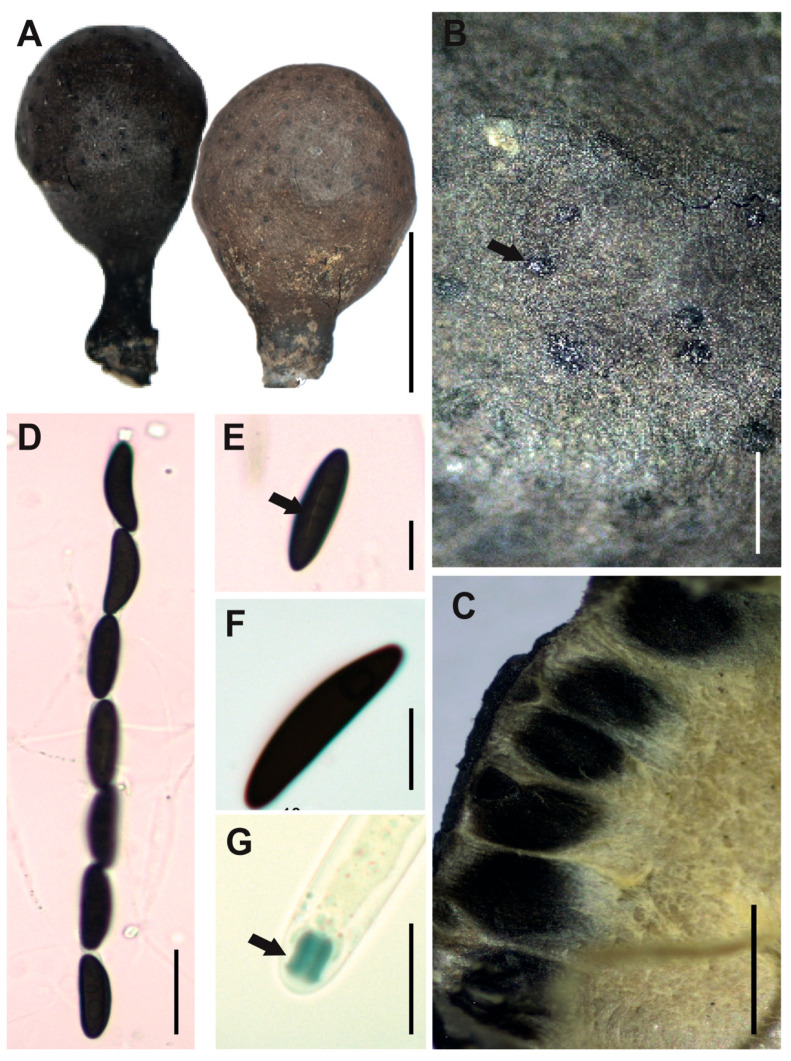
*Xylaria tuberoides* HUTPL(F)-1559. (**A**) External macroscopic characteristics of dry ascoma. (**B**) Black dots of superficial perithecia with slightly papillate ostioles (black arrow). (**C**) Transversal section showing black perithecia immersed into the stroma. (**D**) Ascus with seven out of eight ascospores (brown) in KOH. (**E**,**F**) Ascospores with partial ventral germ slit (black arrow). (**G**) Amyloid apical apparatus (black arrow) in Meltzer reagent. Scale bars: 1 cm (**A**); 1 mm (**B**); 0.5 mm (**C**); 20 μm (**D**); 10 μm (**E**–**G**).

**Remark.** *Xylaria tuberoides* is morphologically described with globose and clavate stromata, close to our specimen [HUTPL(F)-1559] (Figure 11) which is globose to subglobose, however the morphological variability in the fruit bodies within the same species of *Xylaria* is common, especially in different development states. Regarding the microscopic structures, our specimen [HUTPL(F)-1559], with elliptical ascospores elliptical, inequilateral ascospores [(24–)28(–32) × (6–)7(–11) μm], is similar to the ascospores described for *Xylaria tuberoides* by Cruz (2015) [84] 24–29 × 7–9 μm, both featuring a partial ventral germ slit—slightly visible in our specimen (Figure 11) and inconspicuous in the original description. Additionally, both share a subglobose, stromatal shape.

Phylogenetically, the ITS-5.8S sequence of specimen HUTPL(F)-1559 (*Xylaria tuberoides*) forms a well-supported clade (Figure 1) together with other sequences identified as *X. tuberoides*, including KP133545 [33], GU300074 [20], and AB376736 partial LSU (Figure S1) [71]). Based on this phylogenetic congruence, we maintain the identification of our specimen as *Xylaria tuberoides*.

## 4. Discussion

Fungal species diversity is high in tropical forests, such as the Estación Científica San Francisco in the Andes, a recognized biodiversity hotspot [35,86]. It is likely that this tropical rainforest harbors thousands of fungal species, as has already been reported by molecular fungal diversity studies from orchid roots [87,88] including ascomycetes. This high richness is consistent with patterns observed in other megadiverse regions, such as the tropical and mixed forests of Guizhou, Yunnan, and Guangxi in China, where Xylariales exhibit high diversity and endemism while fulfilling key ecological roles as decomposers of decaying wood and fallen branches [89]. In this study, 15 of 20 xylaroid specimens (Table 1) were assigned to ten morphological distinct species *Xylaria adscendens*, *X.* cf. *anisopleura*, *X. apiculata*, *X. curta*, *X. enterogena*, *X. fissilis*, *X. globosa*, *X.* aff. *telfairii*, *X. tuberoides*, and *X. aenea*, the latter representing a new record for Ecuador. While the species *X*. cf. *anisopleura*, *X*. aff. *telfairii*, and *X. aenea* are partially supported by our molecular data, the phylogenetic analysis presented here (Figure 1 and Figure S1) serves as a preliminary systematic positioning of these specimens within the genus rather than a global reconstruction. This local approach complements broader multigene studies (e.g., [19,20,89]) by providing detailed morphological and molecular data from a Neotropical locality often underrepresented in international databases. Despite the scarcity of reference sequences for the LSU region (Figure S1) which precluded a more refined phylogeny, the ITS-5.8S marker proved more informative (Figure 1), as discussed below.

Correspondence between morphological and molecular data.

*Xylaria adscendens* [specimen HUTPL(F)-1448] matches well with several published descriptions of the species based on ascospores morphology and measurements [30,75,76,77,78]. Additionally, our ITS-5.8S sequences are cluster with published sequences (Table 2) GU322432 by Hsieh [20] and KP133298 from Ecuadorian cloud forest reported by Thomas [33], both identified as *X. adscendens* (Figure 1). However, our LSU sequence is cluster together with one sequence identified as *Xylaria curta* (JF773598; Figure S1), isolated as an endophyte from *Taxus globosa* [90] that probably represents a misnamed sequence, which is unfortunately common [91].

The *Xylaria aenea* [HUTPL(F)-1543] is proposed here as a new record for Ecuador, as no previous validated reports of this species have been found in the available literature. For example, Lodge [92] reported numerous *Xylaria* species for cloud and high montane forest in six Neotropical countries (Belize, Ecuador, the Guianas, Mexico, Puerto Rico, and Venezuela), but *X. aenea* was not included for Ecuador. Similarly, Thomas [33] did not describe *X. aenea* in their study, though their unidentified “*Xylaria* sp. strain 931” matches our specimen at the ITS locus (KP133516). Furthermore, our ITS-5.8S sequence for *X. aenea* (Figure 1 and Figure S1) did not cluster with any sequences of well-described species available in the GenBank database or in published studies (Table 2), suggesting that reference sequences for this species are currently lacking.

The continuous study of biodiversity hotspots is essential for uncovering global fungal richness. This is exemplified by recent research in Asia, where intensive surveys documented *Xylaria frustulosa*, *X. glebulosa*, and *X. longissima* as new records for the Chinese mainland, significantly expanding the known geographical distribution of these taxa [93]. Our discovery of *X. aenea* in the Andean foothills similarly contributes to bridging these distributional gaps in the Neotropics. However, molecular evidence indicates a close affinity with *X. enterogenea*. According to Ju and Hsieh [79], some specimens of *X. enterogenea* were probably misclassified as *X. aenea* because of overlapping morphological features.

*Xylaria* cf. *anisopleura* [specimen HUTPL(F)-1458] is closely related to the sequence KP133317, obtained from strain 938 (Ecuadorian cloud forest) identified as *Xylaria anisopleura* [33]. Comparisons based on the LSU D1/D2 region show that the specimen clusters with sequence AB376732, derived from an axenic culture of an ascocarp identified as *X. anisopleura* [71]. Given the inconclusive evidence—specifically, the morphology (Figure 4) and the ITS-5.8S and LSU D1/D2 sequences (Figure 1 and Figure S1)—specimen HUTPL(F)-1458 is tentatively identified as *Xylaria* cf. *anisopleura*. In a recent study by Forin [19], this clade is treated as part of the *X. anisopleura* complex.

The species *Xylaria anisopleura* and *X. globosa* based on their highly variable morphology are difficult to differentiate, as extensively discussed by Fournier [85]. However, our specimens HUTPL(F)-1497 and HUTPL(F)-663 are considered as *X. globosa* due to their asexual state exhibiting a red exudate, a typical morphological character for this species. Moreover, phylogenetic analysis of the ITS-5.8S region (Figure 1) shows that our sequences cluster with other identified as *X. globosa* (e.g., KP133429; [33]) and are genetically distinct from *Xylaria* cf. *anisopleura* [HUTPL(F)-1458]. These results are consistent with those reported by Forin [19], who recovered both species in separate clades based on ITS-5.8S and multilocus analyses (ITS, ACT, TUB2, and RPB2).

Specimens [HUTPL(F)-581 and HUTPL(F)-704] are proposed as *Xylaria apiculata* based on morphological characteristics (see remark) and the phylogenetic ITS-5.8 sequences placement (Figure 1). These specimens clustered with two sequences (accession number KP133325 and KP133335) from ascomata collected in the Ecuadorian cloud forest, previously identified as *X. apiculata* [33]. However, one ITS-5.8S sequence (EF026149) obtained from stroma identified as *X. venosula* and GU300090 identified as *X. arbuscula* also grouped within the same clade as *X. apiculata*. This suggests that the two species might be synonymous, as proposed by Dennis [80]. On the other hand, when compared to our *X. apiculata* LSU sequences (Figure S1), one sequence (JQ760898) identified as *X. arbuscula* by U’Ren [74] appears closely related. However, morphologically, *X. arbuscula* differs from *X. apiculata* primarily in its shorter, branched stromata and smaller ascospores (typically less than 16 × 6 μm), *sensu* Dennis [80]. These species appear to be genetically and morphologically related, as noted by Hsieh [20] who proposed the *Xylaria arbuscula* aggregate. This group includes *X. arbuscula*, *X. arbuscula* var. *plenofissura*, *X. bambusicola*, *X. striata*, and *X. venosula*, all sharing common characteristics such as a “pointed apex on cylindrical stromata and a striped, persistent peeling layer on the stromatal surface”.

Based on morphology (see remark) and phylogenetic placement (ITS-5.8S and LSU), specimen HUTPL(F)-1532 is identified as *Xylaria curta*. This identification is supported by the similarity of our specimen to other *X. curta* sequences, such as KP133352 and MG768837, obtained from axenic cultures in the Ecuadorian cloud forest by Thomas [33], as well as the fruitbody QCAM4545 reported by Guevara [59] (Table 2). In the LSU phylogeny, a closely related sequence identified as *Xylaria feejeensis* (AB376696) clusters within the *X. curta* clade, which may indicate a close genetic relationship or a possible misidentification. Both species are considered part of the *X. corniformis* aggregate and share morphological features such as a “finely cracked outer stromatal layer, wrinkled surface, and ascospores mostly 8–16 μm in length” [19].

In this study, we identified specimen HUTPL(F)-1437 as *Xylaria enterogena* based on morphological characteristics and ITS-5.8 sequence analysis. Our sequence clusters with other sequences (KP133370, KP133371, and KP133372) from strains isolated from ascomata collected in the Ecuadorian cloud forest and identified as *X. enterogena* by Thomas [33]. While *X. enterogena* HUTPL(F)-1437 shares similar ascospore shape and measurements with *Xylaria* aff. *telfairii* HUTPL(F)-1083, these two species are genetically distinct.

The specimen HUTPL(F)-1083 is suggested as *Xylaria* aff. *telfairii* due to their macro and microscopical similarity to *Xylaria telfairii* reported by Dennis [80], and Rogers [31]. Genetically, our sequence of *Xylaria* aff. *telfairii* is closely related to other sequences (KP133541, KP937370) identified as *Xylaria telfairii* from ascomata collected in the Ecuadorian cloud forest by Thomas [33]. However, the genetic difference among these sequences exceeds 3% based on pairwise distance of the ITS-5.8S region. This level of divergence is also evident in the ITS-5.8S phylogenetic tree presented by Forin [19], where the sequence from HUTPL(F)-1083 is nevertheless retained under the name *Xylaria telfairii*. Both species, *Xylaria enterogena* and *X. telfairii* had been discussed as related species that could overlap the shape and measurements of ascospores [31,80].

Specimens HUTPL(F)-1514, HUTPL(F)-1542, and HUTPL(F)-1436 are proposed as *Xylaria fissilis* based on morphological similarities and ITS-5.8S sequence clustering with KP133404, a sequence from an *Xylaria fissilis* isolate collected in the Ecuadorian cloud forest by Thomas [33].

*Xylaria tuberoides*, specimen HUTPL(F)-1559, is supported by the phylogeny (Figure 1). Sequences from HUTPL(F)-1559 cluster with KP133545 [isolated from ascomata in the Ecuadorian cloud forest by Thomas [33], GU300074 (from ascomata, Hsieh [20]), and AB376736 partial LSU (Figure S1) from axenic culture, by Okane [71].

This study provides a baseline of morpho-molecularly characterized species, contributing significantly to our understanding of fungal diversity and their ecological roles. All *Xylaria* spp. encountered in this study were saprotrophic on decaying wood, confirming their well-established role as decomposers of organic matter [1,2,94], thereby contributing to nutrient recycling in the forest.

It is likely that many *Xylaria* species represent cryptic lineages, a phenomenon increasingly documented across the fungal kingdom [95]. Consequently, further research on *Xylaria* species, including examination of herbarium and type specimens, is necessary to comprehend the diversity within this genus, as outlined by Chen [23]. Future studies should incorporate additional molecular markers, such as beta-tubulin [16,20] to analyze intra- and inter-specific variability in depth, thereby establishing a robust barcode gap for accurate species delineation.

In this study, the observed incongruence between morphology and nrDNA ITS-5.8S and LSU sequences likely reflects the high intragenomic polymorphism and paralogous copies characteristic of *Xylaria* [20]. As demonstrated in other groups such as *Golovinomyces* [96], these polymorphisms often lead to sequence overlaps between distinct species, complicating identification via ribosomal markers alone. This biological complexity explains the partial resolution of our nrDNA ITS-5.8S and LSU data and reinforces the necessity of adopting multi-locus frameworks in future research. By integrating these molecular findings with exhaustive morphological evidence, our results establish a taxonomic baseline that facilitates the inclusion of Neotropical specimens into broader evolutionary frameworks, connecting local inventories with global multigene phylogenies [19,20,89,93].

Adhering to the guidelines of the “Outline of Fungi and fungus-like taxa” [97], which seeks to stabilize fungal classification and prevent incorrect taxonomic inferences, our study places these species within the Xylariaceae (Xylariales). This family remains one of the most diverse within the Sordariomycetes, with *Xylaria* being a notably speciose genus that accounts for a significant portion of the order’s diversity, currently estimated to exceed 500 species globally [97]. The high diversity found in this preliminary survey—comprising 10 species and 4 additional phylotypes—reflects the global pattern of "speciose genera" where intensive local sampling continues to reveal hidden richness, especially in under-documented Neotropical regions. To date, scientific records from the Estación Científica San Francisco have been limited to specific groups, including approximately 75 mycorrhizal fungi [37] and three morphologically distinct *Tulasnella* species [39,98], making this contribution a vital expansion of the area’s known mycobiota.

## Figures and Tables

**Figure 5:**
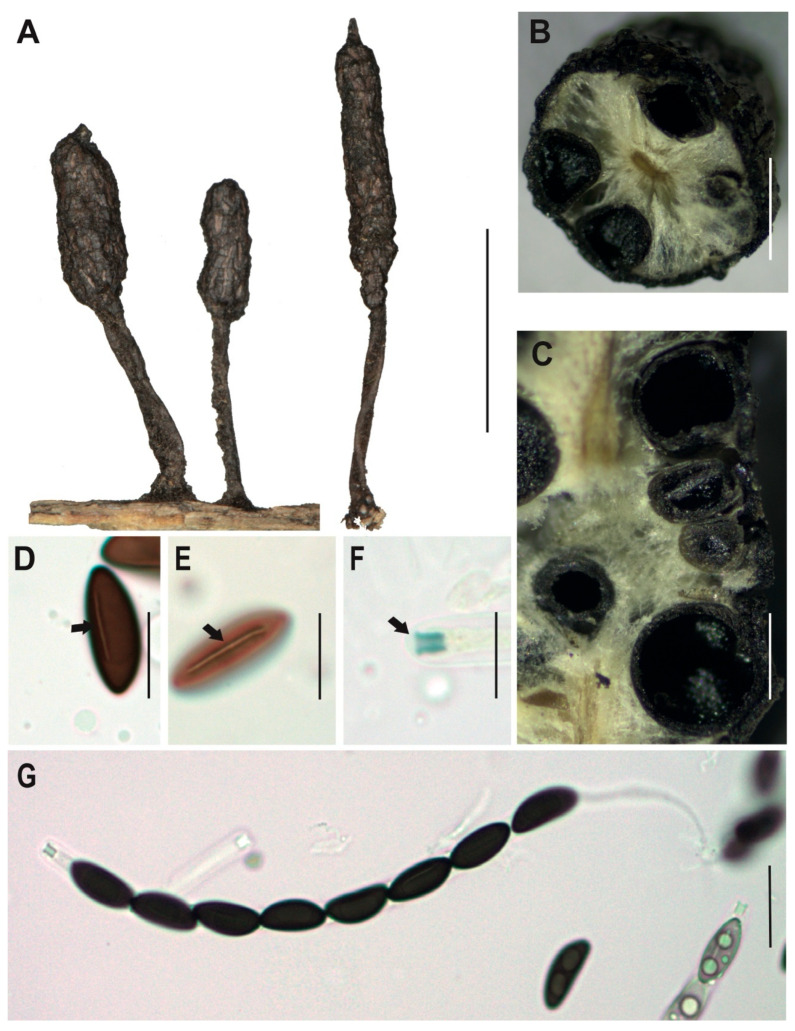
*Xylaria apiculata* [HUTPL(F)-581]. (**A**) External macroscopic characteristics of the ascocarp. (**B**,**C**) Longitudinal and transversal section showing the black perithecia totally immersed into the stroma. (**D**,**E**) Ascospores, partial ventral germ slit (black arrows). (**F**) Amyloid apical apparatus (black arrow) at the ascus tip. (**G**) Ascus with eight ascospores in KOH. Scale bars: 1 cm (**A**); 1 mm (**B**); 0.4 mm (**C**); 10 μm (**D**–**F**); 20 μm (**G**).

**Figure 8:**
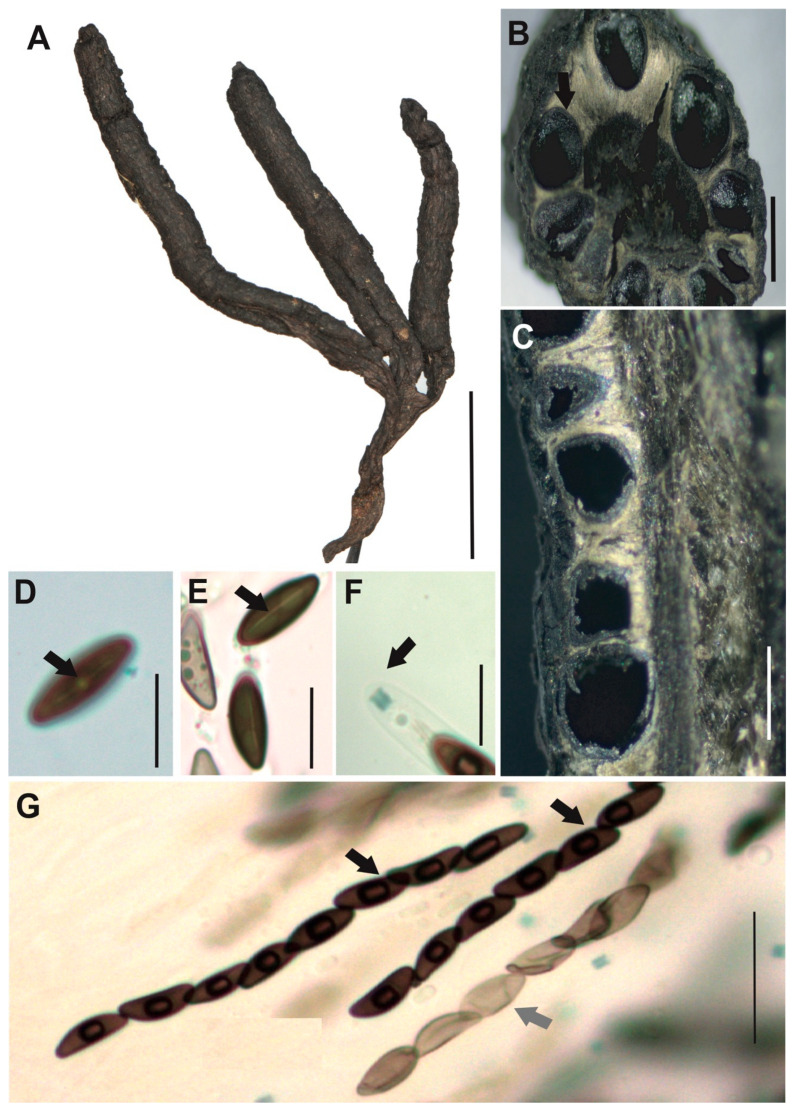
*Xylaria fissilis* [HUTPL(F)-1542]. (**A**) external macroscopic characteristics of the ascocarp and stipe. (**B**) Transversal section showing the black perithecia with papillate ostiole (black arrow) around the stroma and central black hollow in the ascocarp. (**C**) Longitudinal section showing the black perithecia totally immersed into the stroma. (**D**,**E**) Ascospores showing the germ slit (black arrows). (**F**) Amyloid apical apparatus at the tip of the ascus (black arrow). (**G**) Asci showing mature brown ascospores (black arrows), immature ascospores (grey arrow) and amyloid apical apparatus to Melzer’s reagent (Blue dots). Scale bars: 1 cm (**A**); 1.5 mm (**B**,**C**); 10 μm (**D**–**F**); 20 μm (**G**).

**Table 1:** Specimens of *Xylaria* studied, and species defined by morphological and molecular data.

No.	Fungarium Code	Determination	ITS-5.8S and LSU D1/D2 (Accession Numbers)
1	HUTPL(F)-604	*Xylaria adscedens* *	OR086092 only ITS-5.8S
2	HUTPL(F)-1448	*Xylaria adscedens*	OR088119
3	HUTPL(F)-1543	*Xylaria aenea*	OR088132
4	HUTPL(F)-1458	*Xylaria* cf. *anisopleura*	OR088123
5	HUTPL(F)-581	*Xylaria apiculata*	OR088126
6	HUTPL(F)-704	*Xylaria apiculata*	OR088127
7	HUTPL(F)-1532	*Xylaria curta*	OR088133
8	HUTPL(F)-1437	*Xylaria enterogena*	OR088131
9	HUTPL(F)-1436	*Xylaria fissilis*	OR088122
10	HUTPL(F)-1514	*Xylaria fissilis*	OR088121
11	HUTPL(F)-1542	*Xylaria fissilis*	OR088120
12	HUTPL(F)-663	*Xylaria globose* *	OR086093 only ITS-5.8S
13	HUTPL(F)-1497	*Xylaria globosa*	OR086091 only ITS-5.8S
14	HUTPL(F)-1083	*Xylaria* aff. *telfairii*	OR088130
15	HUTPL(F)-1559	*Xylaria tuberorides*	OR088134
16	HUTPL(F)-603	*Xylaria* sp. *	OR086094 only ITS-5.8S
17	HUTPL(F)-921	*Xylaria* sp. *	OR088128
18	HUTPL(F)-1077	*Xylaria* sp. *	OR088124
19	HUTPL(F)-1100	*Xylaria* sp. *	OR088125
20	HUTPL(F)-1533	*Xylaria* sp. ^	Sequence not available

* Anamorph state; assigned to phylogenetic species based on molecular data derived from nrITS-5.8S and LSU D1/D2 sequences. ^ Anamorph state without DNA information.

## Data Availability

The original contributions presented in this study are included in the article/Supplementary Material. Further inquiries can be directed to the corresponding author.

## Supplementary Materials

The following supporting information can be downloaded at: https://www.mdpi.com/article/10.3390/jof12030211/s1, Figure S1. Phylogenetic tree LSU partial region with new sequences (highlight in color) from *Xylaria* spp. from Southern Ecuador. Values at the nodes correspond to Maximum likelihood BS (left) and BPP (right), respectively. Only values > 50% and 0.5 are shown on nodes. Tree was rooted with *Parahypoxylon papillatum* (NG_066379).
